# Variations in the Size and Shape of Human Cochlear Malformation Types

**DOI:** 10.1002/ar.24136

**Published:** 2019-04-24

**Authors:** Anandhan Dhanasingh

**Affiliations:** ^1^ MED‐EL GmbH Innsbruck Austria

**Keywords:** inner ear malformation, 3D visualization, size and shape variation, cochlear height, basal turn diameter

## Abstract

The objective of this study is to determine the variations in size and shape of the most widely recognized cochlear malformation types using three‐dimensional (3D) visualization. Using 3D slicer freeware, the complete inner‐ear structures were segmented from 46 anonymized high‐resolution computed tomography (HRCT) image datasets. Cochlear height, internal auditory canal height, and width were measured from the axial plane. Cochlear basal turn diameter was measured from the oblique coronal plane. Number of cochlear turns was measured from the 3D images and the corresponding cochlear duct length (CDL) was estimated using the CDL equations given in Alexiades et al. [Otol Neurotol 36 (2015) 904–907]. Out of 46 preoperative HRCT image datasets of human temporal bone, cochlear anatomy types including normal anatomy (4), enlarged vestibular aqueduct syndrome (3), cochlear aplasia (2), incomplete partition Types I (8), II (Mondini's deformity) (3), and III (X‐linked) (4), cochlear hypoplasia (CH) (17), and common cavity (CC) (5) were identified. Majority of CH cases had cochlear height shorter than 4 mm whereas the CC cases measured cochlear height above 6 mm. For all the other malformation types, cochlear height was between 4 and 6 mm. In terms of “A” value, majority of CH cases showed shorter “A” value of <7.5 mm, which is in the lower end in comparison to the rest of the malformation types reported in this study. 3D‐visualization shows the size and shape variations of all the structures of inner ear and also improves the clinicians' ability to visualize cochlear anatomy and nearby structures much easier than from the 2D image slices. Anat Rec, 302:1792–1799, 2019. © 2019 The Author. *The Anatomical Record* published by Wiley Periodicals, Inc. on behalf of American Association for Anatomy

As per literature reports, approximately 20% of the sensorineural hearing loss (SNHL) population have some degree of malformed cochlear–vestibular anatomy (Sun et al. 2015; Sennaroğlu and Bajin 2017). Enlarged vestibular aqueduct syndrome (EVAS), incomplete partition (IP) Types I, II (Mondini's), III (X‐linked), cochlear hypoplasia (CH), common cavity (CC), and cochlear aplasia (CA) were the most widely reported malformation types (Sennaroglu and Saatci 2002).

Normal anatomy cochlea has been studied enough to understand the fact that the size and shape variation is significant (Meng et al. 2016; Thong et al. 2017). As a result, supporting preplanning tool like OtoPlan (www.otoplan.ch) is now European Confirmity marked and is in clinical application (Lovato and de Folippis 2019) assisting clinicians in measuring the cochlear size and choosing the cochlear implant (CI) electrode accordingly. Though abnormal anatomy cochleae were studied enough with the help of 2D radiographic images (Sennaroglu and Saatci 2002; Sun et al. 2015; Sennaroğlu and Bajin 2017), only limited number of reports (Hara et al. 2012; Liu et al. 2017; Booth et al. 2018) are available bringing most of the widely recognized malformation types especially in three‐dimensional (3D) format in knowing the variation in its size and shape.

Cochlear implantation is the ultimate solution to restore hearing in the SNHL population (Dhanasingh and Jolly 2017) and for relatively inexperienced CI surgeons, mental compilation of series of 2D images in to 3D representation of anatomical structures might be challenging. This article attempts to capture the outer morphology of the complete inner‐ear structures including the internal auditory canal (IAC) in 3D format. Also, the scope of this study is extended to capture the variations in size, shape, and the number of cochlear turns among the most widely reported cochlear–vestibular malformation types.

## MATERIALS AND METHODS

**Figure 1:**
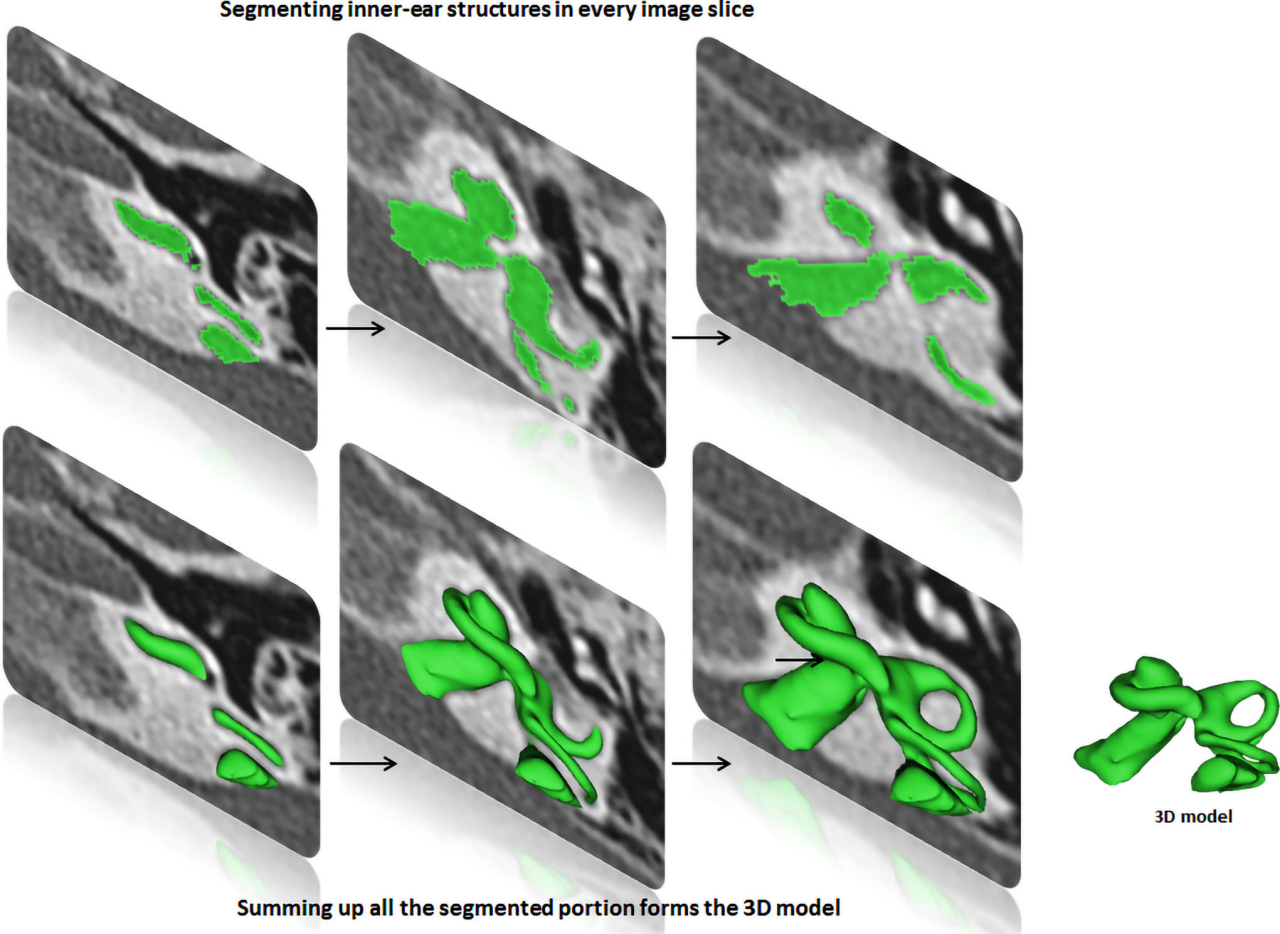
Segmentation of the inner ear from every image slice available in the image dataset. Summing up all the segmented images transforms into a 3D model.

Forty‐six preoperative high‐resolution computed tomography (HRCT) image datasets of anonymous human temporal bones with variety of cochlear–vestibular malformations were shared by several clinics across the world from the year 2011 to 2018 for educational purposes. The image data sets were loaded into 3D slicer freeware (3D Slicer, https://www.slicer.org/; version 4.8.0) followed by segmentation of the complete inner‐ear structures including the IAC. Axial plane is better suitable for segmentation of these structures by setting a tight gray scale threshold to avoid capturing undesired structures (refer Fig. 1). Figure 1 elaborates the segmentation process. Approximately 10 min was needed to segment the complete inner ear including the IAC from clinical imaging dataset.

**Figure 2:**
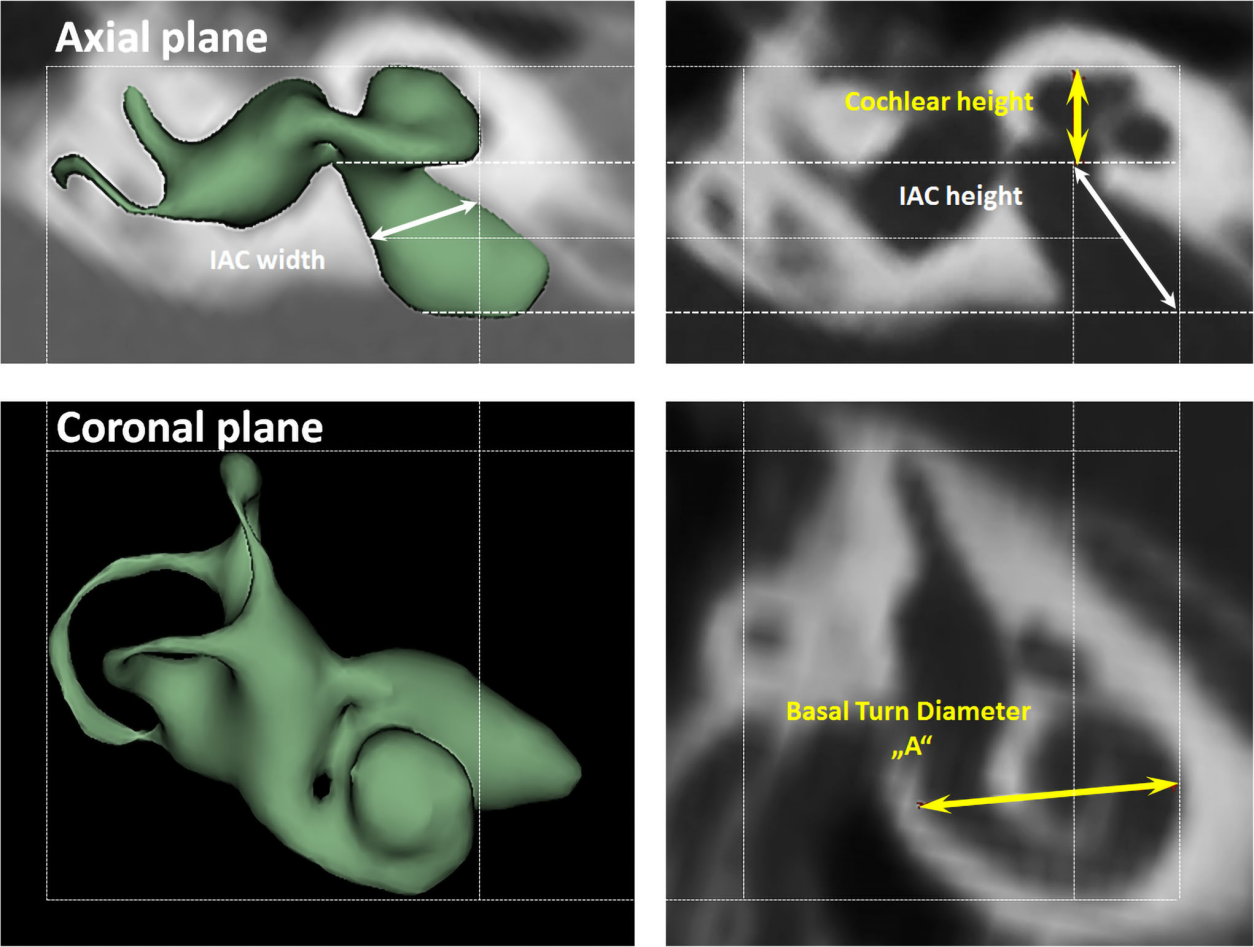
Basal turn diameter of the cochlea “A” measured from the oblique coronal plane. Cochlear height, IAC height, and the IAC width were all measured from the axial plane.

Cochlear height was measured in the axial plane, from the lower most surface of the basal turn to the upper most surface of the apex. Basal turn diameter “A” value was measured in the oblique coronal plane starting from the round window entrance passing through the mid‐modiolar section to the opposite side of the lateral wall as shown in Figure 2. Height and width of IAC were measured in axial plane as shown in Figure 2. Number of cochlear turns were approximated from 3D images and corresponding cochlear duct length (CDL) was estimated using CDL equations given else where (Alexiades et al. 2015).

## RESULT

From the 46 preoperative HRCT image dataset of the human temporal bones, cochlear anatomy types including normal anatomy (4 ears), EVAS (3 ears), CA (2 ears), IP Types I (8 ears), II (Mondini's deformity) (3 ears), and III (x‐linked) (4 ears), CH (17 ears), and CC (5 ears) were identified.

**Figure 3:**
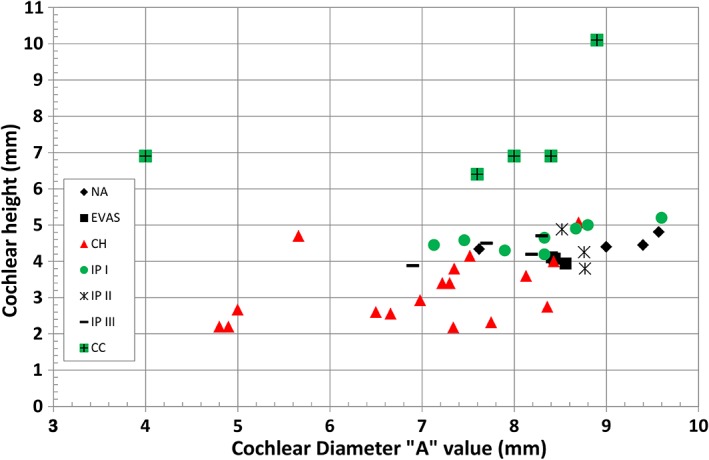
Cochlear height and the cochlear basal turn diameter measured from all the image data set. Legends correspond to the different malformation types.

Figure 3 summarizes cochlear height, cochlear basal turn diameter “A,” height, and width of IAC measurements for the malformation types identified in this study (Table 1).

**Table 1:** List of malformation types identified along with the number of cases under each type

Anatomy type	No. of cases per anatomical type
Normal anatomy (NA)	4
Cochlear aplasia (CA)	2
EVAS	3
IP Type I	8
IP Type II with EVAS	3
IP Type III	4
Cochlear hypoplasia (CH)	17
Common cavity (CC)	5
Total no. of image sets analyzed	46

In terms of cochlear height and cochlear basal turn diameter “A,” CH group was clearly distinguishable from the rest of other malformation types by having its height ≤4 mm for majority of the cases and the “A” <8.5 mm. On the other hand, the heights of all CC cases were well above 6 mm. Height of NA, EVAS, IP Types I, II, and III were between 4 and 5 mm. “A” values of these malformation types were between 7 and 9.6 mm.

**Figure 4:**
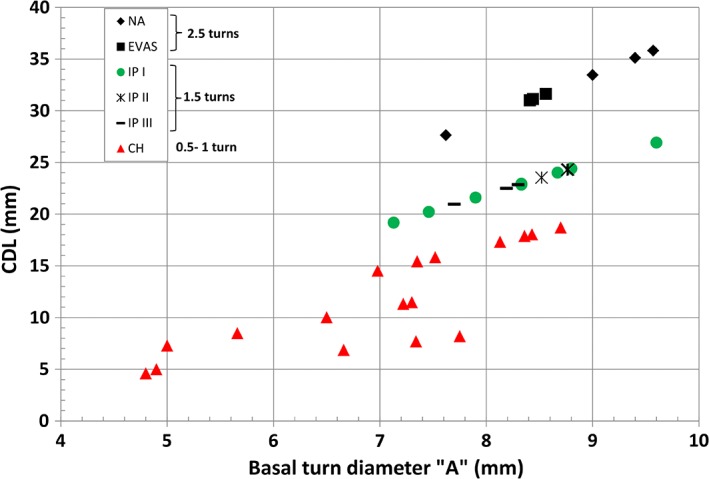
Number of cochlear turns and corresponding estimated CDL.

While NA and EVAS cochlear types have typically 2½ turns, which is equal to 900‐degree angle of angulation, IP Types I, II, and II typically showed 1½ turns of cochlea which is only 540‐degree angle of angulation. CH showed a variation in number of turns from 0.5 to 1 and in terms of angulation this equals 180‐degree angle to 360‐degree angle. The corresponding CDLs were estimated using mathematical CDL equations given in Alexiades et al. (2015) taking the “A” value as input and the results are given in Figure 4. NA and EVAS cochlear types had CDL well above 27 mm and extended up to 36 mm. IP Types I, II, and III estimated CDL from 19 mm to a maximum of 27 mm. Estimated CDL for CH ranged from 4.5 mm to a maximum of 19 mm, which was on the lower range in comparison to the other malformation types.

**Figure 5:**
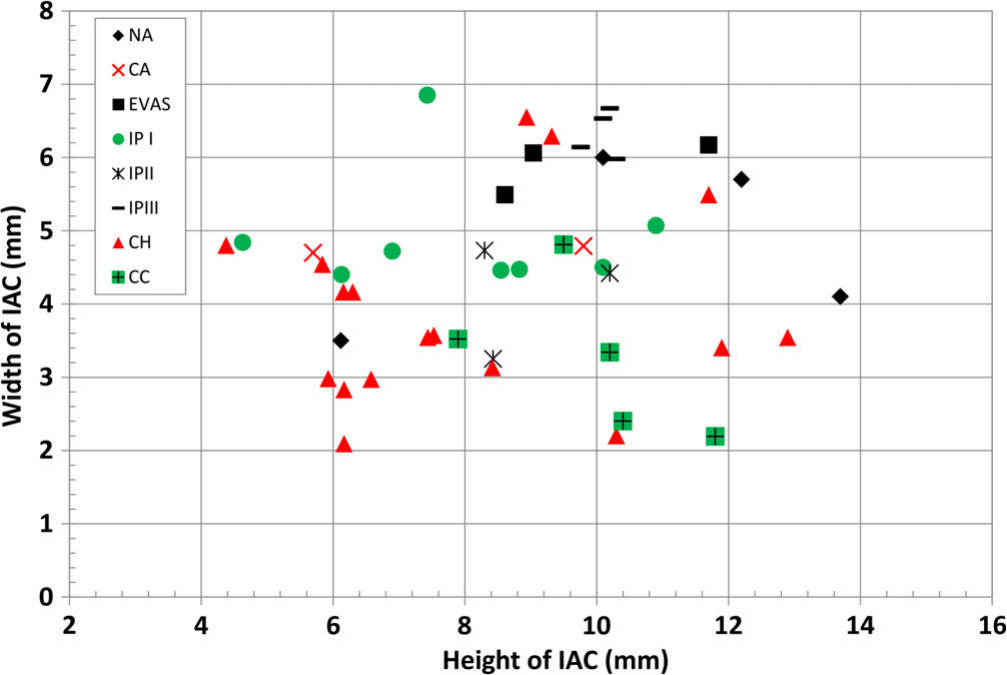
Height and width of IAC for all the cochlear malformation types reported in this study.

Size of IAC measured by its length and width in axial plane showed a great variation among the malformation types reported in this study. Figure 5 with height of IAC in the *x*‐axis and width of IAC on the *y*‐axis showed that none of the cochlear malformation types can be clearly distinguishable. Overall, the length of the IAC varied between 4 and 14 mm whereas the width varied from 2 to 7 mm.

Outer shape of the complete inner ear for all the malformation types reported in this study is given in Table 2 in the form of 3D images both in axial and as well in coronal view.

**Table 2:** 3D models of the most widely reported malformation types

S. No	Anatomy type	S. No	Anatomy type
Normal anatomy (NA)			
NA_1 (L)	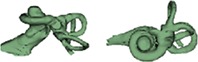	NA_3 (R)	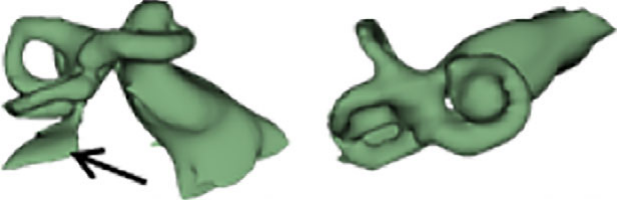
NA_2 (R)	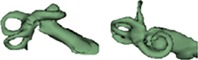	NA_4 (R)	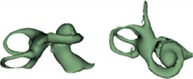
Cochlear aplasia			
CA _5 (L) no measurements	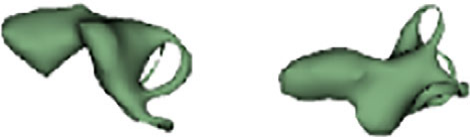	CA _6 (R) no measurements	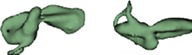
Enlarged vestibular aqueduct syndrome (EVAS)			
EVAS_7 (R)	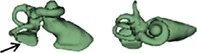	EVAS_9 (L)	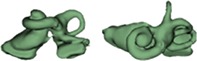
EVAS_8 (L)	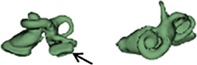
Incomplete partition Type I (IP Type I)			
IP I_10 (R)	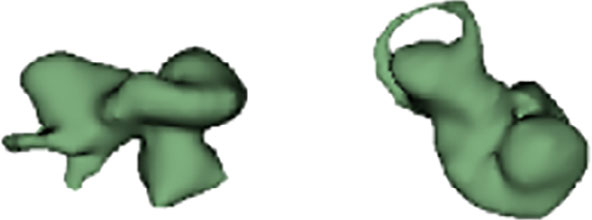	IP I_14 (L)	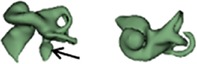
IP I_11 (R)	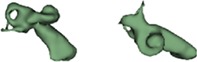	IP I_15 (R)	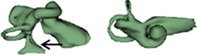
IP I_12 (L)	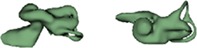	IP I_16 (L)	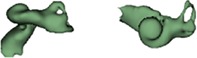
IP I_13 (L)	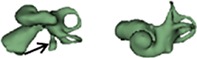	IP I_17 (R)	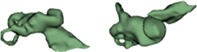
Incomplete partition Type II (IP Type II)			
IP II_18 (L)	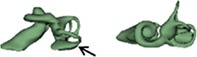	IP II_20 (L)	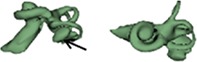
IP II_19 (L)	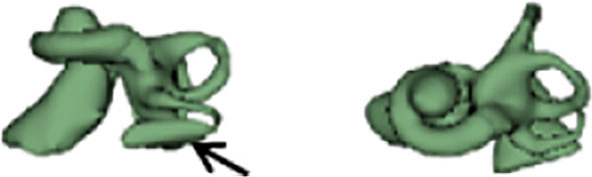
Incomplete partition Type III (IP Type III)			
IP III_21 (R)	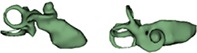	IP III_23 (L)	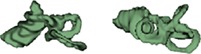
IP III_22 (L)	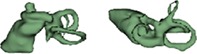	IP III_24 (R)	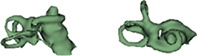
Cochlear hypoplasia (CH)			
CH _25 (L)	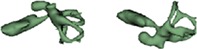	CH_34 (R)	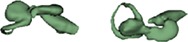
CH_26 (R)	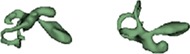	CH_35 (R)	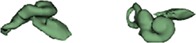
CH_27 (L)	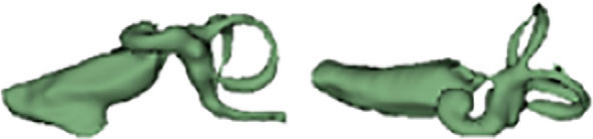	CH_36 (L)	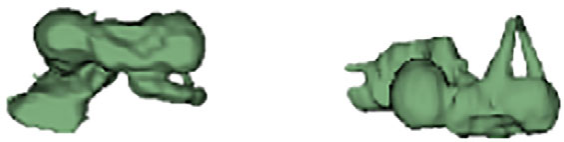
CH_28 (L)	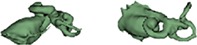	CH_37 (L)	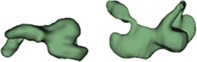
CH_29 (R)	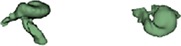	CH_38 (L)	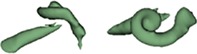
CH_30 (L)	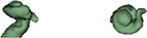	CH_39 (R)	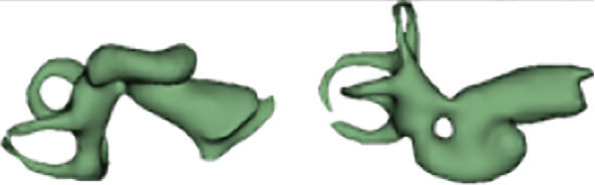
CH_31 (L)	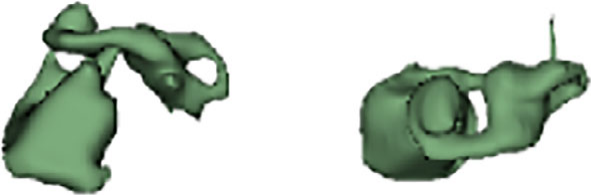	CH_40 (R)	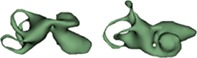
CH_32 (L)	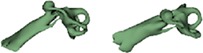	CH_41 (R)	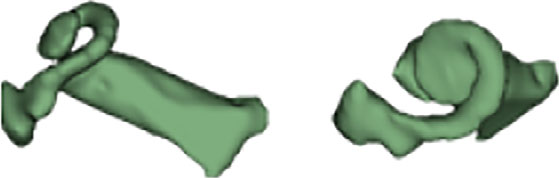
CH_33 (R)	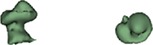		
Common cavity (CC)			
CC_42 (R)	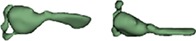	CC_45 (R)	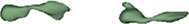
CC_43 (L)	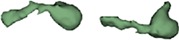	CC_46 (R)	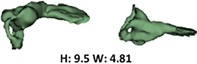
CC_44 (L)	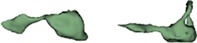	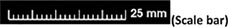	

S. No. refers to the overall number of samples along with the malformation types. L/R in brackets refers to left and right side ear.

All images are in the same scale.

The normal anatomy cochleae (1–4) showed well‐developed cochlear lumen along with vestibular structures. Sample 3 showed normal presence of vestibular aqueduct (VA) pointed by the black arrow. CA samples (5, 6) showed complete absence of cochlea but with somewhat normal presence of the vestibular structures. EVAS (7–9) had almost normal presence of cochlear and vestibular structures but the VA was pretty much enlarged (thickness >1.5 mm) as pointed by the black arrow. IP Type I (10–17) had varying size and shape of the cochlear, vestibular, and IAC. Samples 13–15 showed enlarged VA as pointed by the black arrow marks. The IP Type II (18–20) had basal turn availability close to one full turn and remaining apical portion appeared cystic. All the IP Type II samples had enlarged VA (black arrow) in this series with its thickness >1.5 mm. IP Type III (21–24) had wider IAC and the cochlea appears very narrow after the basal turn. CH (25–41) showed the most variation in terms of size and shape of the complete inner ear. Samples 25–27, 32, 34, 36, 37, and 39 had only ½ of the basal turn. Samples 29–31, 33–38, and 41 had either partial presence or complete absence of the vestibular structures. CC malformation types (42–46) had higher degree of variation in size and shape of the cavity that combines both cochlea and vestibular part. While samples 42–44 had only one Semicircular canals (SCC), sample 45 had no SCCs. Sample 46 showed all three SCCs.

**Figure 6:**
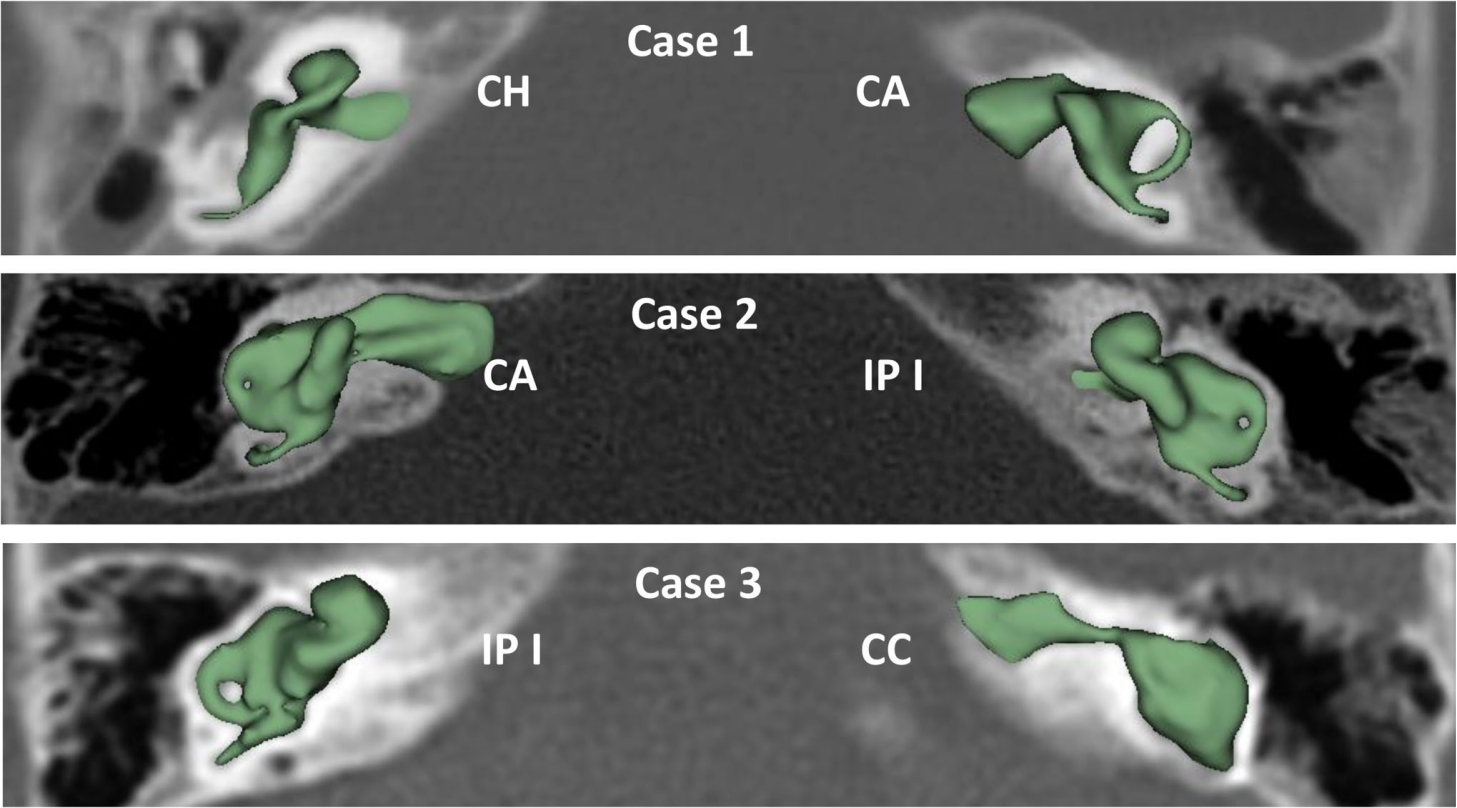
Dissimilar cochlear malformation types on either side of the ears.

Dissimilar cochlear malformation types were identified in three cases as shown in Figure 6. Case 1 showed CH on right side and CA on left side. Case 2 had CA on right side and IP I on left side. With case 3, it was IP I on right side and CC on left side.

## DISCUSSION

Figures 3, 4, 5 and Table 2 summarize overall size variation in terms of cochlear height, cochlear basal turn diameter, height and width of IAC, and their shape variation in 3D for all the cochlear–vestibular malformation types identified in this study. Liu et al. (2017) demonstrated the variation in cochlear size (height and basal turn diameter) in 2D images from 110 patients covering the malformation types of aplasia, CC, IP Types I and II, and CH. In their study, they have shown that CH had shorter cochlear height and “A” value, which was reflected in our study as well as shown in Figure 3. In their study, the IP Type II group merged well with normal anatomy group and this was also reflected in our study though the sample number was small. What was not covered in their study was IP Type III and EVAS, which was covered in the current study. The CDL estimated using the CDL equations for the number of cochlear turns available from each of the malformation types showed a great variation (Fig. 4). The shorter CDL estimated within the CH types should not be mixed up with the normal anatomy cochlea with shorter CDL as reported by Grover et al. (2018). Yiin et al. (2011) covered nicely most of the widely reported cochlear–vestibular malformation types from the preoperative 2D radiographic images but not in 3D as given in this study. Sennaroğlu and Bajin (2017) and Sennaroglu (2016) showed the various malformation types in 2D radiographic and histological slices. Though these 2D images and histological slices are educational in the identification of various malformation types, nothing related to the cochlear dimensions and 3D images were reported in that study.

Adibelli et al. (2017) came out with a new way of classifying cochlear malformation types using INCAV system covering all the inner‐ear structures (**I**nternal auditory canal, cochlear **N**erve, Cochlea, vestibular **A**queduct, and **V**estibule) using the MRI. Applying INCAV system in our study was not possible due to the availability of HRCT only from which the detection of cochlear nerve is not possible. Also, the VA was not detected clearly in all image datasets in our study.

From the 46 image datasets analyzed, CH type malformation was identified in 17 datasets showing the prevalence of this malformation type from the dataset available in this study. This is the type that also showed great variation in overall size and shape of inner ear and also in the number of SCCs of the vestibular portion. Dissimilar malformation types on either side of ears are not uncommon and this need to be considered when comes to cochlear implantation.

For experienced CI surgeons who have treated good number of patients with cochlear malformation may be able to detect malformation types directly from preoperative 2D radiographs. With the new/young CI surgeons coming to the field, identifying malformation types correctly from 2D radiographs might be challenging. The proposed 3D segmentation of complete inner ear from preoperative HRCT images allows us to get the 3D image of inner ear in a matter of 10 min, which is worth spending in better understanding on the severity of the anatomical malformation.

## CONCLUSIONS

It is mentally a challenging task for many clinicians to compile a series of 2D image slices into an accurate 3D representation of any anatomic structure. A clear 3D understanding of inner‐ear anatomy with pathological conditions will be an advantage to any surgeon undertaking a complex procedure. The large variations in size and shape of inner ear and in number of SCCs shown in this study are yet another proof confirming that every cochlea is unique in its appearance and anatomy. 3D visualization, as demonstrated in this study, improves the clinicians' ability to visualize cochlear anatomy and nearby structures much easier than from 2D image slices.

## CONFLICT OF INTEREST

Author is employed by MED‐EL GmbH as Topic Manager for Electrodes, which is purely a scientific role with no marketing activities.
